# Assessing detector stability and image quality of thermal cameras on smartphones for medical applications: a comparative study

**DOI:** 10.1007/s11517-025-03348-4

**Published:** 2025-04-04

**Authors:** Vladan Bernard, Erik Staffa, Jana Pokorná, Adam Šimo

**Affiliations:** Department of Biophysics, Faculty of Medicine, Masaryk University, Kamenice 3, 625 00 Brno, Czech Republic

**Keywords:** Infrared thermography, Smartphone-based thermal imaging, Temperature stability, Surface temperature accuracy, Spatial uniformity

## Abstract

**Introduction:**

Infrared thermography (IRT) has gained significant interest in medical applications for its potential in diagnosing various conditions. Smartphone-based IRT modules offer portability and affordability, leading to increased utilization in medical settings. However, differences in performance among these modules raise questions about their reliability for medical use.

**Materials and methods:**

This study compared three smartphone-based IRT modules (SmartIRT—Hikmicro, FLIR One Pro, and Seek Thermal CompactPRO—which, according to their datasheets, exhibit comparable quality and parameters. Temperature stability, surface temperature of the body, and spatial uniformity of provided images were assessed using calibrated black body measurements and surface temperature monitoring.

**Results:**

The Hikmicro module exhibited the most stable temperature readings, while FLIR One Pro showed the highest temperature increase over time. Seek Thermal CompactPRO demonstrated relatively better spatial uniformity. However, discrepancies in image resolution were noted, with FLIR One and Seek modules modifying image sizes through post-processing algorithms.

**Conclusion:**

While SmartIRT modules offer affordability and portability, their performance varies significantly. Temporal stability emerges as a critical factor, with the Hikmicro module demonstrating leadership in this aspect. Careful consideration and validation are necessary when selecting and utilizing SmartIRT modules for medical applications.

**Graphical abstract:**

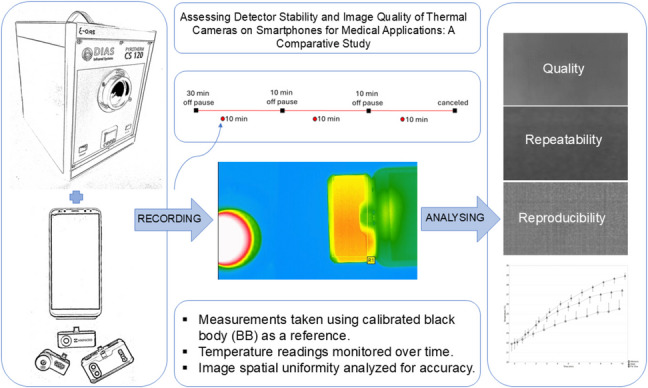

## Introduction

Over the past several decades, growing interest in thermal infrared imaging technology has led to extensive exploration of its use in various fields of medicine. Continuous development primarily focused on accuracy and image resolution has enabled researchers to find new perspective in the diagnosis and follow-up of both common and rare medical conditions. These include ischemia, arthritis, fever, tumours such as melanoma and several others [1].

Major advancements in infrared imaging have led to miniaturization of devices, making the technology both more affordable and accessible to medical professionals. Affordable smartphone-based thermal cameras (SmartIRT) such as FLIR ONE (FLIR Systems, Wilsonville, OR) have been compared to a high-performance standalone FLIR camera in diagnosing diabetic foot ulcers. One study demonstrated high agreement in measurements between the two types of cameras, with the SmartIRT device achieving a 1% higher sensitivity (94%) and 5% lower specificity (86%) in diagnostic accuracy compared to the high-performance IR camera [2].

The key question is whether all SmartIRT devices can be used for medical purposes, as is often suggested in scientific articles. The popularity of using these microdevices for surface temperature measurements has increased due to the rising number of scientific publications in recent years.

For example, FLIR ONE SmartIRT was used to measure skin temperature at burn wounds sites in five patients as an important clinical marker for assessing both wound depth and prognosis for treatment [3]. When compared to indocyanine green laser angiography, a method with double the diagnostic accuracy of a standard clinical assessment [4], the non-salvageable area of the burn wound measured by FLIR ONE overlapped by an average of 91%, with only a minor underestimation of the salvageable area margins.

A large number of studies have used FLIR ONE camera as a tool for measuring surface temperature in connection with COVID-19 disease [5, 6]. Various studies have examined its potential for detecting COVID-19 based on surface temperature. Brzezinski et al. developed with positive results an automated image processing algorithm for the analysis of thermal images of the back. Their thermal imaging analysis was inversely correlated with clinical variables associated with COVID-19 disease progression [7].

Martinez‐Jimenez et al. used the SmartIRT to detect COVID-19 in minimally symptomatic individuals. Unfortunately, their findings indicated that absolute temperature recordings could not distinguish between infected and non-infected individuals [8]. This correspond with a review by Khaksari et al., which reported on the unreliability of IRT due to poor sensitivity and specificity in detecting true core body temperature and its inability to identify asymptomatic carriers [9]. Ma et al. and Jiang et al. used FLIR ONE in the connection with COVID-19, primarily to develop improved image processing methods. The first group of authors aimed to achieve safe and accurate temperature measurements even when a person’s face was partially obscured. They ultimately proposed a cloud-edge-terminal collaborative system with a lightweight infrared temperature measurement model [10]. The second group proposed an abnormal breathing detection in subjects connected with COVID-19 based on combining both method RGB and thermal videos [11]. Both research teams utilized the FLIR ONE camera in their studies.

Although the FLIR ONE may appear to be the current standard for the clinical use of SmartIRT devices, several other manufacturers have introduced similar devices that offer high-resolution IR imaging and are powered solely by the smartphone’s internal battery, providing significant convenience.

One such devices is the SEEK compact PRO, manufactured by the US-based company Seek Thermal. It offers a thermal imaging resolution of 320 × 240 pixels, double that of the FLIR ONE’s 160 × 120 pixels. A study by Kirimtat et al. compared the performance of two SmartIRT devices in assessing a toe injury. The results showed that although the sensors of both devices had identical thermal sensitivity (70mK) and could therefore distinguish minor temperature differences with the same accuracy, the SEEK compact PRO was better suited for the close-up imaging (less than 45 cm), due to its adjustable focal length, a feature absent in the FLIR ONE. However, image quality was found to be better in the latter device, largely due to the better image processing and better suited thermographs enabling to distinguish contours of the subject more easily [12].

Improvements in both image quality and resolution undoubtedly play a crucial role in the next step of IR imaging utilization in clinical applications: deep learning. In a recent study by Al Husaini et al., researchers combined IR imaging and deep learning to develop a smartphone application as a detection tool for early breast cancer. The self-diagnostic tool uses basic information inputted by the user, a thermogram from the FLIR ONE Pro, and a deep learning algorithm to determine whether the user should consider visiting a medical specialist. The study showed that the app achieved a 100% accuracy rate in breast cancer detection with the uncompressed image. However, images with higher compression rates, reflected in lower image quality, led to a slight drop in accuracy to 99.9% [13].

The undeniable advantage of these devices includes their low acquisition cost and often simple user interface and software. However, can we fully rely on the data they provide. One important question is whether all SmartIRT devices of a similar level, as declared by their manufacturers, truly provide comparable quality or whether significant differences exist that necessitate choosing a specific manufacturer to guarantee measurement accuracy. One way to assess their performance is to compare them with each other under similar conditions. Our focus was not to determine whether micro-devices are suitable for medical temperature measurement applications (we assume that the resolution of the IRT detector in these devices is sufficient for the given type of measurement) but rather on assessing the accuracy and stability of measurements, particularly when comparing the different devices available on the market.

Thus, the main objective of this study is to compare portable microIRT devices that can be connected to a smartphone. One of the most commonly used in scientific studies (the FLIR ONE) was included in the measurements, along with less frequently used devices, as identified through a full-text search in the Web of Science (WoS) database.

## Materials and methods

### SmartIRT devices

Three different smartphone-compatible IRT modules were tested and compared in our measurements. All three SmartIRT devices can be connected and operated using a smartphone or tablet.

The first device tested was the Hikmicro model HM-TJ11-3AMF-Mini1 (Hangzhou Microimage Software Co., Ltd., Zhejiang, China). This device features a focal plane array microbolometer thermal sensor with a resolution 160 × 120 (19,200 pixels in total) and a thermal sensitivity 40 mK. The SmartIRT module operates with a minimum fixed focus distance of 20 cm to infinity. According to the manufacturer’s technical specifications, the system has a frame rate of 25 Hz. The device features a USB-C connector and is powered by the smartphone. The HIKMICRO Viewer software (Hangzhou Microimage Software Co. Ltd, Zhejiang, China) was used with Hikmicro IRT module.

The second SmartIRT device tested was the FLIR ONE Pro (FLIR Systems, Inc., Wilsonville, USA). This module has a focal plane array microbolometer thermal sensor with resolution of 160 × 120 pixels and thermal sensitivity 70 mK. It operates with a fixed focus distance of 15 cm to infinity. According to the manufacturer’s technical specifications, the system has a frame rate of 8.7 Hz. The device features a USB-B connector and is powered by its own internal battery. The FLIR One version 2.2.5 software (FLIR Systems Aktiebolag, Wilsonville, USA) was used with the FLIR One Pro module.

The third SmartIRT module tested was the Seek Thermal CompactPRO (Seek Thermal, Inc., Santa Barbara, USA). This device features a focal plane array microbolometer thermal sensor with a resolution of 320 × 240 pixels and a thermal sensitivity of 70 mK. The SmartIRT module operates with an adjustable focus distance of 15 cm to infinity. According to the manufacturer’s technical specifications, the system has a frame rate of 9 Hz. The device features a USB-B connector and is powered by the smartphone. The Seek Thermal software (Seek Thermal, Inc., Santa Barbara, USA) was used with the Seek Thermal CompactPRO module. The SmartIRT modules were connected to smartphones Vivo Y70 (v2023) and Huawei RNE-L21.

### Measurement of body surface temperature of SmartIRT modules

The body surface temperature of the SmartIRT modules was recorded by using the VarioCAM HD infrared camera (InfraTec GmbH, Dresden, Germany). This camera features an uncooled microbolometer focal plane array detector with a resolution of 640 × 480 pixels and a spectral range of 7.5 to 14 μm. According to the manufacturer, the absolute accuracy of the measurement is declared as ± 2 °C or ± 2% of the reading, with a thermal sensitivity of 30 mK. The IRBIS 3.1 Professional software (InfraTec GmbH, Dresden, Germany) was used with the VarioCAM HD IRT device to evaluate the data in the “Evaluation of temporal and spatial uniformity of SmartIRT recorded images” section.

The obtained data were processed in Excel (Microsoft Corporation, Redmond, Washington) and statistically analysed using Statistica 12 (Microsoft Corporation, Redmond, Washington) software.

The ImageJ 1.53t software was used for plot profile measurement.

A PYROTHERM CS 120 infrared calibration model (black body BB, Dias Infrared GmbH, Dresden, Germany) was used as a stable temperature source set to 37 °C. The calibration source has a temperature range of − 15 to 120 °C, an emissivity of 0.98 ± 0.01, an uncertainty of ± 0.5 K, and a control stability of ± 0.2 K.

All experiments were conducted under the same temperature conditions. The ambient temperature was 22 °C, and no external thermal radiation sources (except for the black body) were present.

### Temperature stability assessment of SmartIRT

The black body (BB) temperature of 37 °C was measured from a distance of 0.6 m, with the SmartIRT device fixed on a tripod in a consistent position for all measurements.

The measurements were conducted following these steps:

1. The SmartIRT and smartphone were allowed to acclimatize to room temperature for 30 min with power turned off. The BB was heated to 37 °C, requiring 8 min according to the manufacturer’s manual.
2. The SmartIRT and smartphone were powered on, and the stable 37 °C temperature of the BB surface was recorded continuously from 0 to 10 min.
3. The SmartIRT and smartphone were then powered off completely for 10 min.
4. This entire measurement cycle was repeated three times.

The data are presented in Fig. 3 as box plots depicting the BB temperature over time, with readings taken at 60-s intervals for 10 min. Temperature readings were recorded every 60 s, starting at 0 min and continuing for a total of 10 min, resulting in 11 measurements.

### Measurement of surface temperature of the rear case of SmartIRT

**Fig. 1:**
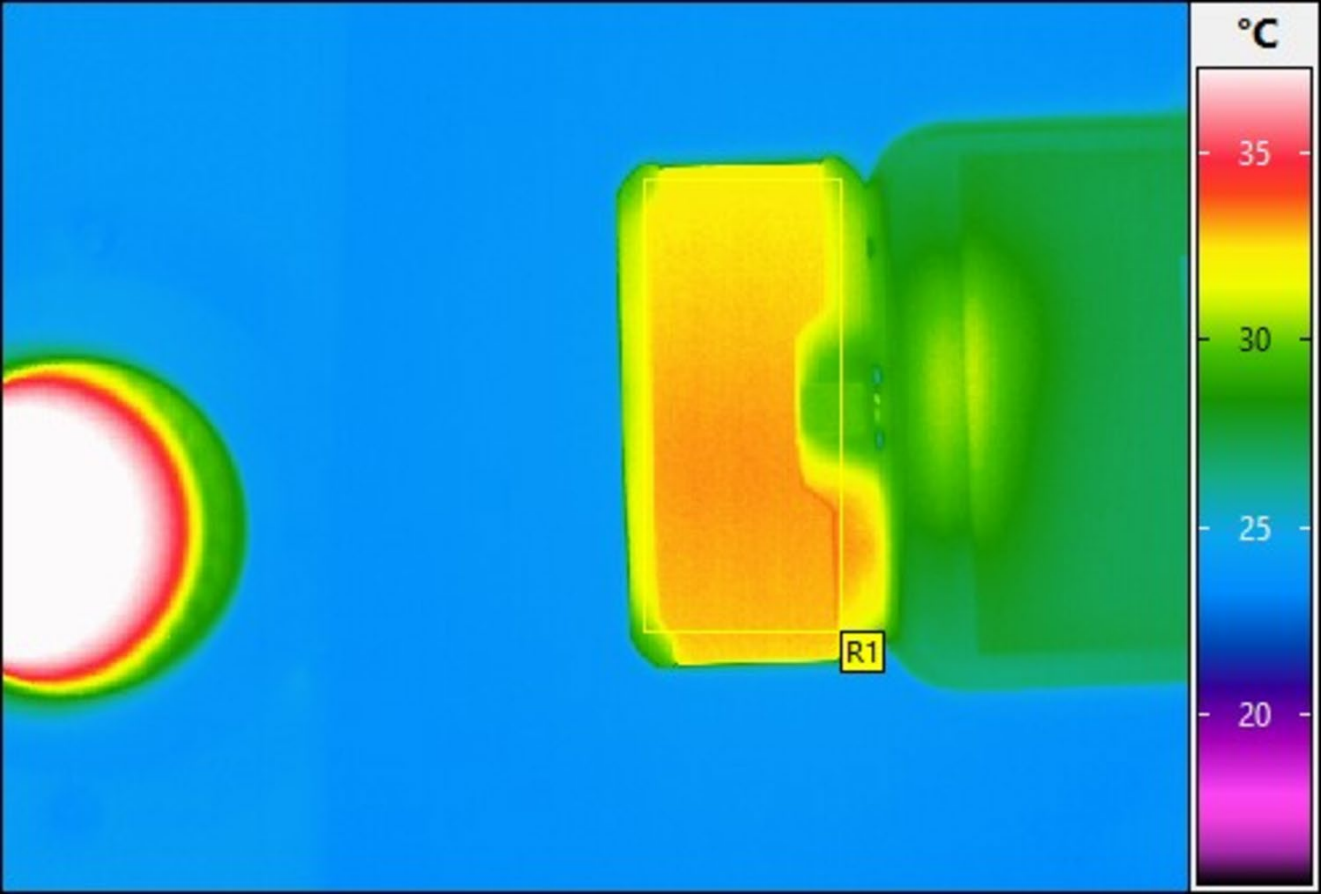
The ROI for measuring body surface temperature of SmartIRT, the maximum temperature was recorded

The region of interest (ROI) on the SmartIRT was chosen the rear side case of SmartIRT. This area was measured using VarioCAM HD infrared camera. The maximum temperature within the ROI was recorded and analysed using the “square sampling” tool, as shown in Fig. 1.

The measurement protocol was similar to that in the “Temperature stability assessment of SmartIRT” section and followed these steps:

1. All devices were allowed to acclimatize to room temperature for 30 min with the power turned off.
2. The devices were then powered on, and the temperature of the ROI on SmartIRT device recorded continuously from 0 to 10 min.
3. The devices were powered off completely for 10 min.
4. This entire measurement cycle was repeated three times for each SmartIRT device.

The data are presented in Fig. 4 showing the increase in maximum temperature in the ROI over time relative to the start of the measurement (0 min). Temperature readings were recorded every 60 s, starting at 0 min and continuing for a total of 10 min, resulting in 11 measurements.

### Evaluation of temporal and spatial uniformity of SmartIRT recorded images

To measure the uniformity of the temperature distribution in the infrared image, a homogenic gypsum board coated with ThermaSpray 800 (TMV SS spol. s.r.o., Prague, Czechia) white paint, with a defined emissivity of 0.96, was used.

The measurements were conducted as follows:

1. All devices were allowed to acclimatize to room temperature for 30 min with power turned off.
2. The devices were powered on and used to measure the gypsum board, which was at a stable with ambient temperature of 22 °C, for 10 min.
3. The devices were powered off completely for 10 min.

For evaluation, three thermal images from each SmartIRT device were selected at 0 min, 2 min, and 10 min. These images were converted to grayscale in the respective IRT software. A diagonal line profile across the entire SmartIRT image was then obtained graphically using ImageJ 1.53t software. The results are presented as plots, showing grayscale level versus distance (in pixels).

Throughout the entire measurement period, the IRT devices remained fixed in the same position relative to the board.

### Summary of experiments

**Fig. 2:**
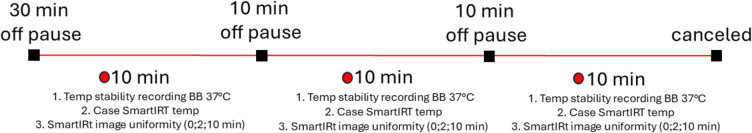
Timeline for the experiments

The measurement process is summarized and shown on the timeline in the Fig. 2.

1. Temperature stability assessment: Measuring the SmartIRT devices’ ability to detect a stable 37 °C temperature over time.
2. Surface temperature measurement: Evaluating how the SmartIRT devices’ own surface temperature changes over time.
3. Uniformity assessment: Analysing the temperature distribution in SmartIRT images to assess temporal and spatial uniformity.

## Results

Three SmartIRT devices underwent a comparative analysis to evaluate their thermal stability, body surface temperature characteristics, and the temporal and spatial uniformity of the SmartIRT images. These parameters are important in determining the suitability of each device for potential medical applications.

### Calibration model BB—temperature stability

**Fig. 3:**
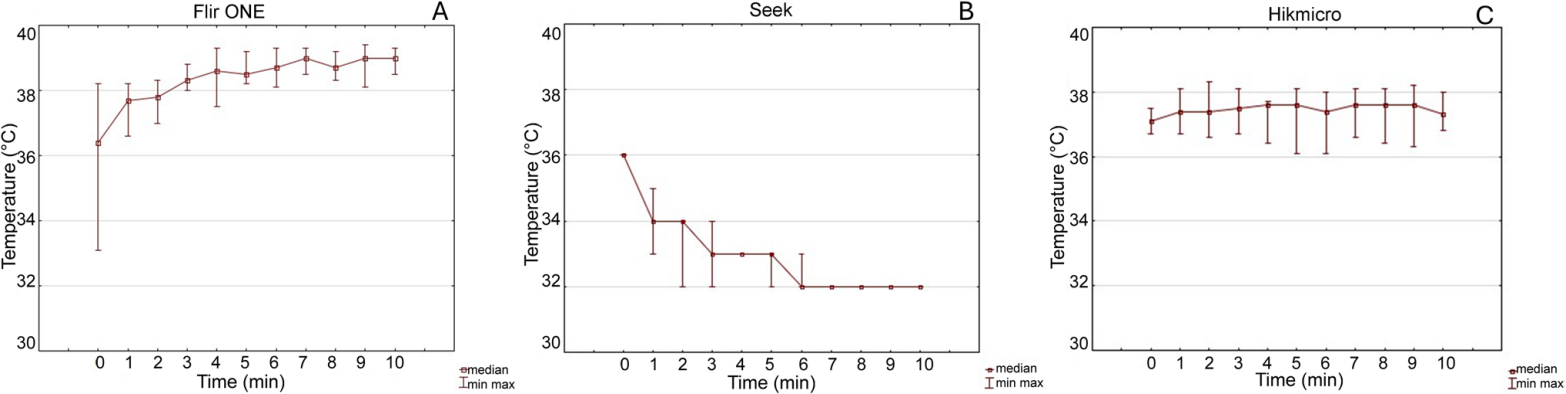
The graph illustrates the temperature stability of the SmartIRT modules (**A**) FLIR One, (**B**) Seek, (**C**) Hikmicro when measuring the black body (BB) calibration source set at 37 °C. The x-axis represents time (in minutes) over a 10-min interval, while the *y*-axis represents temperature and boxplots represent median value, minimal and maximal value for each time of snapshot

A BB calibration source was maintained at a constant temperature of 37 °C for this analysis. The graphs (Fig. 3) illustrate the temperature variations recorded by the SmartIRT devices over a 10-min period. The data highlight the differences in temperature accuracy and stability among the tested SmartIRT devices over time. The results show a gradual temperature increase over time for all three devices, with the FLIR One exhibiting the highest recorded temperatures, followed by Seek Thermal and Hikmicro.

### Body temperature measurement of SmartIRT modules

**Fig. 4:**
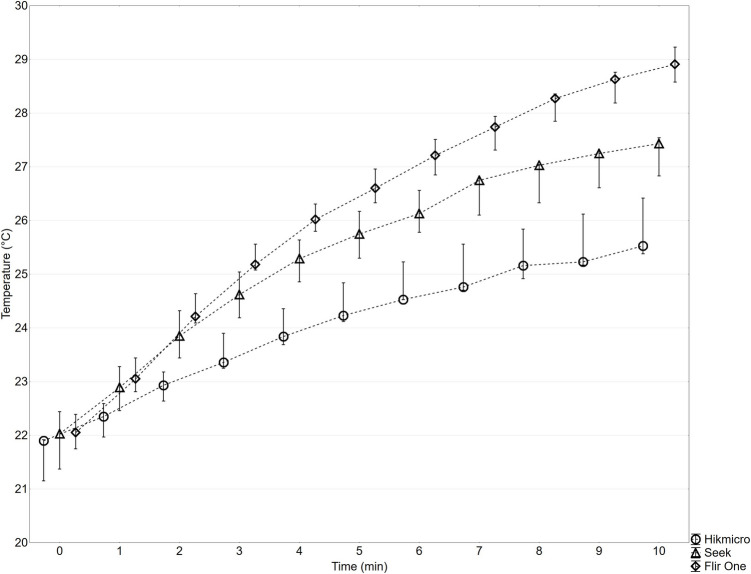
The graph presents the time-dependent temperature increase of the rear casing of three different SmartIRT modules (Seek, Hikmicro, and FLIR One) over a 10-min operational period. The *x*-axis represents time (minutes), while the *y*-axis represents temperature and boxplots represent median value, minimal, and maximal value

Figure 4 presents the body surface temperature profiles of the SmartIRT modules as measured using the VarioCAM HD IRT camera. The ROI represents the temperature progression at the rear of the SmartIRT module casing over 10-min interval.

The most temperature rise was observed in the FLIR One module. After 10 min of operation, the temperature increased by 7 °C for FLIR One, 5 °C for Seek, and 4 °C for Hikmicro. These findings indicate that the FLIR One module exhibited the highest surface heating, while the Seek and Hikmicro modules showed relatively lower temperature increases during operation.

### Evaluation of temporal and spatial uniformity of SmartIRT image

One of the objectives of this study was to assess the uniformity of SmartIRT image. The SmartIRT devices were used to measure the temperature of a surface with uniform characteristics, including temperature, texture, and emissivity (a gypsum board painted with ThermaSpray 80). The resulting SmartIRT images were then analysed in grayscale. The analysis involved extracting plot profiles along a diagonal slice across each image, extending from one corner to the opposite. Ensuring uniformity in SmartIRT images is crucial for achieving accurate and consistent temperature measurements, which is essential for medical and other thermal imaging applications.

It is important to note that measurement results are influenced by factors such as the thermal stability and sensitivity of the sensor chip, as well as by the different post-processing mechanisms applied within the SmartIRT software.

**Fig. 5:**
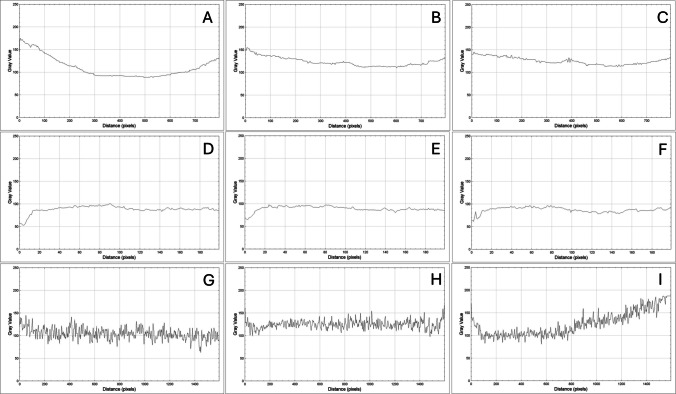
Plot of profiles extracted from the full diagonal of SmartIRT image over time. **A**, **B**, **C** FLIR One in 0, 2, and 10 min. **D**, **E**, **F** Hikmicro in 0, 2, and 10 min. **G**, **H**, **I** Seek in 0, 2, and 10 min

**Fig. 6:**
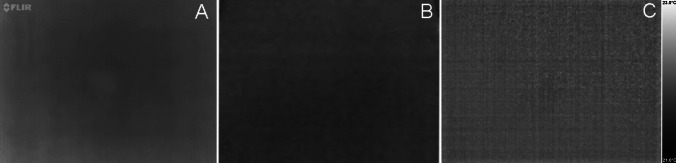
Thermal image of board under identical measurement conditions from FLIR One (**A**), Hikmicro (**B**), and Seek (**C**) in grayscale palette

Temperature stability across the entire image area (i.e., spatial uniformity) is not maintained, as shown by the data in Fig. 5. Ideally, the curve should be with minimal fluctuation in the *y*-axis, indicating uniform temperature distribution. However, deviations are observed among the devices. FLIR One exhibits a symmetrical temperature drop in the SmartIRT image, centred around the middle. Hikmicro does not display a symmetrical temperature shift, and local maxima and minima frequently appear in its plot profiles. The temporal variation in uniformity is particularly prominent in the FLIR One instrument, with noticeable changes over time (Fig. 6).

The pixel count of SmartIRT images obtained from the FLIR One and Seek does not correspond to the native resolution of their sensor chips. The increased pixel count results from post-processing techniques applied by the instrument’s software [14, 15].

The default image resolutions provided by each device are the following:

FLIR One: 640 × 480 pixels; Seek: 1280 × 960 pixels; Hikmicro: 160 × 120 pixels.

These values represent the default (or lowest possible) resolution settings at which the SmartIRT images are output by each device.

## Discussion

This study compared smartphone-based infrared thermography modules (SmartIRT), focusing on their temperature stability, body surface temperature development, and spatial uniformity in providing SmartIRT images. However, the study does not aim to determine whether these devices can fully replace high-end scientific IRT devices. Additionally, it does not provide a detailed comparison of their software capabilities, even though software quality significantly impacts device performance (e.g., automatic post-processing of SmartIRT images). Our results offer insights into the performance and realisability of the Hikmicro, FLIR One Pro, and Seek Thermal CompactPRO, which are increasingly used in medical applications due to their affordability. However, several key factors—temperature stability, surface temperature accuracy, and spatial uniformity—must be carefully considered when evaluating their potential use in medical settings.

To assess temperature stability over time, we used a calibrated black body (BB) as a stable reference temperature source for all three SmartIRT devices. The results revealed different measurement behaviours:

- The Seek Thermal CompactPRO showed a noticeable decrease in the indicated BB temperature, with a median temperature drop of 4 °C over 10 min.
- The FLIR One Pro exhibited a median temperature increase of 2 °C during the same period.
- The most stable device was Hikmicro, with temperature values oscillating around the initial measurement.

Such variations in temperature stability could potentially impact measurement accuracy, particularly in medical applications where precision is crucial. It is well known that the microbolometric sensor’s temperature affects the device’s measurement accuracy and functionality [16]. A measurable difference in the temperature of the sensor during the measurement, thus the temperature of the entire device, will lead to a drift of the indicated temperature, as the sensitivity of the sensor changes [14]. This issue has been extensively studied, and some bolometric IRT devices employ temperature drift compensation mechanisms, such as active cooling, to maintain stability [17]. Given these considerations, we examined the thermal instability of SmartIRT devices during a 10-min continuous operation period. Measuring the body surface temperature of the SmartIRT devices over time revealed differences in temperature rise among the three models. This was expected due to the absence of active cooling. The graph in Fig. 4 shows an almost continuous rise in surface temperature for the FLIR One Pro. Temperature stabilization of the FLIR One Pro device was not reached after a monitoring time period. The observed temperature increase after 10 min of operation was 7 °C for ROI. The Seek and Hikmicro devices exhibited a non-linear increase in body surface temperature, particularly towards the end of the measurement interval. These findings suggest that the FLIR One has less efficient thermal management than other two devices, leading to a higher temperature rise during prolonged use.

Spatial and temporal uniformity of the SmartIRT image is critical, especially in medical applications. Consistent and uniform imaging is essential for repeatability and accuracy in thermal mapping of surfaces.

To evaluate this, all SmartIRT devices were tested on a unirm surface in terms of temperature and structure, completely filling the field of view. The images were then converted to 8-bit grayscale for analysis. The results showed discrepancies in temperature distribution across the image area:

- FLIR One Pro exhibited a temperature drop symmetrically from the centre of the image.
- Hikmicro displayed frequent local maxima and minima, indicating irregularities in temperature distribution.
- Seek Thermal CompactPRO demonstrated better spatial uniformity than the other two modules.

These variations in spatial uniformity emphasize the importance of post-processing algorithms and image calibration in ensuring consistent and accurate thermal imaging. The evaluated SmartIRT images differed in resolution, even though FLIR One and Hikmicro share the same sensor size. Only Hikmicro provided original-resolution images (160 × 120 pixels), whereas FLIR One and Seek applied automatic upscaling. The FLIR One and the Seek images were automatically changed in post processing, the so-called process of upscaling, to the size of 640 × 480 and 1280 × 960 pixels, respectively. The process is known as the multi spectral dynamic imaging (MSX) technology for FLIR [18]. While some authors consider this an advantage, it can also be seen as a limitation. Any image modification without transparency regarding the applied algorithms introduces uncertainty, which is particularly problematic in medical applications [14].

FLIR One exhibits higher temperature variations and stability, which may limit its suitability for precise temperature measurement. Hikmicro and Seek demonstrate more stable performance, though Hikmicro’s lower resolution may affect its precision in certain applications.

Furthermore, it is noteworthy that the pixel count of the SmartIRT images obtained from the FLIR One and Seek modules does not correspond to the native resolution of their thermal sensors. This discrepancy is attributed to post-processing algorithms employed by the software of the modules, which may affect the image quality and spatial resolution.

In conclusion, our study provides valuable insights into the performance characteristics of smartphone-based infrared thermal imaging modules commonly used in medical applications. While these devices offer portability and affordability, their temperature stability, surface temperature accuracy, and spatial uniformity of provided images vary among different models. Thus, careful evaluation and validation are necessary when selecting SmartIRT modules for medical applications. Users typically consider sensor size and noise equivalent temperature difference (NETD) as key indicators of IRT device quality. However, our findings suggest that temporal and spatial stability should also be carefully assessed to ensure repeatability and reliability in diagnostic applications.

## Conclusion

In summary, this study evaluated the performance of three widely available smartphone-based infrared thermal imaging modules suitable for potential use in medical applications. Our findings highlight differences in temporal and spatial temperature stability, developing the body surface temperature in time and spatial uniformity of the images provided among the tested modules. These insights contribute substantially to a deeper understanding of the capabilities and limitations of smartphone-based IRT modules in medical settings.

Despite their similar appearance, these SmartIRT devices exhibit varying levels of temperature measurement quality. While it would be unrealistic to expect them to consistently match the precision of high-end IRT devices, such precision may not always be necessary for practical applications. However, our study indicates that certain SmartIRT modules may be unsuitable for accurate temperature measurement due to inherent quality limitations and specific design choices, even if their small sensor resolution appears acceptable. Notably, our findings do not unequivocally endorse any specific product, challenging the assumption that brand recognition necessarily correlates with superior performance.

Temporal stability in temperature measurement emerges as a critical factor for SmartIRT devices, with the Hikmicro IRT module demonstrating the most consistent performance in this aspect. Additionally, the ability to capture SmartIRT images in their original resolution, without artificial enhancement, stands as a notable advantage of the Hikmicro device.

These findings emphasize the importance of careful selection, validation, and consideration when using SmartIRT modules for medical applications.

## References

[1] Ring EFJ, Ammer K (2012) Infrared thermal imaging in medicine. Physiol Meas 33(3):R33 22370242 10.1088/0967-3334/33/3/R33

[2] Van Doremalen RFM, Van Netten JJ, Van Baal JG, Vollenbroek-Hutten MMR, van der Heijden F (2019) Validation of low-cost smartphone-based thermal camera for diabetic foot assessment. Diabetes Res Clin Pract 149:132–139 30738090 10.1016/j.diabres.2019.01.032

[3] Xue EY, Chandler LK, Viviano SL, Keith JD (2018) Use of FLIR ONE smartphone thermography in burn wound assessment. Ann Plast Surg 80(4):S236–S238 29489530 10.1097/SAP.0000000000001363

[4] Wongkietkachorn A et al (2019) Indocyanine green dye angiography as an adjunct to assess indeterminate burn wounds: a prospective, multicentered, triple-blinded study. J Trauma Acute Care Surg 86(5):823 30589753 10.1097/TA.0000000000002179 PMC6493689

[5] Luze H et al (2021) Personal protective equipment in the COVID-19 pandemic and the use of cooling-wear as alleviator of thermal stress: a pilot study in plastic surgery staff members. Wien Klin Wochenschr 133:312–320 33301061 10.1007/s00508-020-01775-x PMC7727095

[6] Meneses-Claudio B, Nuñez-Tapia L, Alvarado-Díaz W, Mantari AA (2021) Implementation of a thermal image processing system to detect possible cases of patients with COVID-19. Int J Emerg Technol Adv Eng 11:130–139. 10.46338/ijetae1121_15

[7] Brzezinski RY et al (2021) Automated processing of thermal imaging to detect COVID-19. Sci Rep 11(1):17489. 10.1038/s41598-021-96900-9 34471180 10.1038/s41598-021-96900-9 PMC8410809

[8] Martinez-Jimenez MA, Loza-Gonzalez VM, Kolosovas-Machuca ES, Yanes-Lane ME, Ramirez-GarciaLuna AS, Ramirez-GarciaLuna JL (2021) Diagnostic accuracy of infrared thermal imaging for detecting COVID-19 infection in minimally symptomatic patients. Eur J Clin Invest 51(3):e13474 33336385 10.1111/eci.13474 PMC7883263

[9] Khaksari K et al (2021) Review of the efficacy of infrared thermography for screening infectious diseases with applications to COVID-19. J Med Imaging 8(S1):010901–010901 10.1117/1.JMI.8.S1.010901 PMC7995646 33786335

[10] Ma Z, Li H, Fang W, Liu Q, Zhou B, Bu Z (2021), A cloud-edge-terminal collaborative system for temperature measurement in COVID-19 prevention. In IEEE INFOCOM 2021 - IEEE Conference on Computer Communications Workshops (INFOCOM WKSHPS), Vancouver, BC, Canada, 2021, pp 1–6. 10.1109/INFOCOMWKSHPS51825.2021.9484616

[11] Jiang Z, Hu M, Fan L, Pan Y, Tang W, Zhai G, Lu Y (2020) Combining visible light and infrared imaging for efficient detection of respiratory infections such as COVID-19 on portable device. Comput Res Repos. 10.48550/arXiv.2004.06912

[12] Kirimtat A, Krejcar O, Selamat A, Herrera-Viedma E (2020) FLIR vs SEEK thermal cameras in biomedicine: comparative diagnosis through infrared thermography. BMC bioinformatics 21(2):1–10 32164529 10.1186/s12859-020-3355-7 PMC7069161

[13] Al Husaini MAS, HadiHabaebi M, Gunawan TS, Islam MR (2021) Self-detection of early breast cancer application with infrared camera and deep learning. Electronics 10(20):2538

[14] Greenwald W Flir one pro review. PCMag. https://www.pcmag.com/reviews/flir-one-pro (accessed 13.03.2024, 2024)

[15] F Systems “FLIR’s multi-spectral dynamic imaging (MSX) technology: tech note.” https://www.flir.eu/globalassets/industrial/instruments/flir-msx-tech-note.pdf (accessed 13.03.2024, 2024)

[16] Olbrycht R, Więcek B (2015) New approach to thermal drift correction in microbolometer thermal cameras. Quant InfraRed Thermogr J 12(2):184–195

[17] Brazane S, Riou O, Delaleux F, Ibos L, Durastanti JF (2024) Management of thermal drift of bolometric infrared cameras: limits and recommendations. Quant Infr Therm J 22(1):54–69. 10.1080/17686733.2023.2290304

[18] Jaspers MEH, Carrière ME, Meij-de Vries A, Klaessens J, Van Zuijlen PPM (2017) The FLIR ONE thermal imager for the assessment of burn wounds: reliability and validity study. Burns 43(7):1516–1523 28536040 10.1016/j.burns.2017.04.006

